# Editorial: Biomarkers in Leishmaniasis

**DOI:** 10.3389/fcimb.2019.00388

**Published:** 2019-11-12

**Authors:** Eugenia Carrillo, Javier Moreno

**Affiliations:** WHO Collaborating Centre for Leishmaniasis, National Centre for Microbiology, Instituto de Salud Carlos III, Madrid, Spain

**Keywords:** biomarkers, leishmaniasis, diagnostic, vaccine, cure

## Introduction

Leishmaniasis is one of the most deadly, yet neglected, of all tropical diseases (Alvar et al., 2012). The eco-epidemiological characteristics of leishmaniasis render it a complex problem. There are over 20 species of leishmaniasis pathogens transmitted by different species of sand fly (*Phlebotomus* spp.). Transmission may be anthroponotic or zoonotic, the latter entailing different animal reservoirs. Depending on the species of *Leishmania* and the immune response to it, the disease may present as cutaneous leishmaniasis (CL), mucocutaneous leishmaniasis (MCL), visceral leishmaniasis (VL), or post-kala azar dermal leishmaniasis (PKDL) (Burza et al., 2018).

The complexity of leishmaniasis means different strategies are needed if it is to be controlled and eliminated (Matlashewski et al., 2014; Rijal et al., 2019). For example:

- Diagnostic methods need to be faster and simpler, but sensitive and robust, and allow early diagnoses to be made. Follow-up methods are also needed that confirm patient responses and help predict the risk of relapse.
- Efficient therapies for the different forms of the disease need to be developed, both for the immunocompetent and the immunodepressed.
- Methods of controlling animal reservoirs—especially domestic animal reservoirs—need to be developed; alongside better vectorial control these should reduce the transmission of the parasite.
- The capacity of asymptomatic carriers to pass on the disease needs to be examined—an important challenge for control programs.
- A vaccine against the different forms of leishmaniasis needs to be developed; this might be the best means of control and protection, but work is needed on how to immunize most effectively for the lowest cost.

Biomarkers have a central role to play in the above challenges by providing information on patient immune status, the response to treatment, exposure to vectors, the role of animal reservoirs, and the epidemiology of infection, etc. New biomarkers need to be found that will allow the development of tools for assessing the effectiveness of treatments, that can confirm when a cure has been achieved, to identify asymptomatic persons and rates of transmission in endemic areas, to develop rapid, non-invasive tests, and for checking the immune response to experimental vaccines (Ibarra-Meneses et al., submitted).

This Research Topic, entitled “Biomarkers in Leishmaniasis”, is a collection of 19 articles, some of which examine the latest advances in biomarkers of the different types of leishmaniasis, while others report original research into biomarker identification and characterization.

## Diagnostic and Disease Progress Biomarkers

Several articles in the cited collection examine the identification of new biomarkers useful for understanding the pathogenesis of leishmaniasis, and for improving its diagnosis. The clinical complexity and epidemiology of leishmaniasis is a challenge in the identification of biomarkers able to track the progress of the disease. This is made clear by different review articles that focus on its different clinical forms. The work of Brodskyn and Kamhawi on biomarkers of zoonotic VL in Latin America, focuses on humans and dogs, and addresses the need to examine a combination of inflammatory mediators for the development of a tool that distinguishes between the different stages of the disease. They also discuss the use of serum antibodies against the highly immunogenic salivary proteins of *Lutzomyia* as biomarkers of exposure to the vector in humans and dogs.

In their review, Bahrami et al. highlight the scarcity of specific markers for CL. Apart from abnormalities in the delayed hypersensitivity test, in T cell subpopulations, cytokine levels and enzyme (e.g., adenosine deamidase and L-argininase) concentrations, these authors suggest the need to develop analyses based on comparing the transcriptome of the lesion with that of healthy skin (Christensen et al., 2016; Masoudzadeh et al., 2017). The identification of biomarkers able to predict the result of infection by different species of *Leishmania* is also a major challenge in CL (Patino and Ramírez, 2017). For example, the physiopathology of PKDL (which follows VL in some treated patients) is different to that of both VL and CL (Kip et al., 2015), and patients show responses to treatment that are difficult to assess (the lesions can take a long time to heal, thus responses may take time to appear). In their review, Zijlstra indicate that current biomarkers for PKDL lesions are unsatisfactory. Certainly, clinical assessment is subjective and not very precise, and while the parasite load can be determined by qPCR, serological tests such as DAT, rK39 ELISA, and rK39 RDT lack specificity since antibodies may hang over from previous bouts of VL. Moreover, the systemic and skin immune responses are different. Zijlstra also questions whether biomarkers in the blood (such as cytokines or cell populations) properly reflect skin-level changes, and declares that new avenues need to be explored. These might include 3D optical scanning and the undertaking of longitudinal studies that can provide a description of PKDL before, during and after cure.

Dogs play a major role in the transmission of the parasite to humans (Moreno and Alvar, 2002). The present collection therefore also includes an article by Maia and Campino which is exclusively devoted to canine leishmaniasis. This review discusses the latest advances in the identification of biomarkers associated with infection by *L. infantum* in dogs. The early detection and treatment of infected animals is a basic requirement in the control of human VL (Alvar et al., 2004). Canine leishmaniasis has a wide spectrum of manifestations, the result of complex host-parasite interactions (Reis et al., 2010), and these authors conclude that no single biomarker is able to confirm a diagnosis, reflect the effectiveness of treatment, or indicate the infectivity of affected dogs.

In their contribution, Ontoria et al. report the expression of different genes in the spleens of infected and control Balb/c mice, the final aim of their research being to better understand the immunological mechanisms that lead to protection or disease progression, and the identification of associated biomarkers. d'El-Rei Hermida et al. review the histological changes that occur in the spleen in severe VL, and record the events that eventually lead to its destruction. Garde et al. discuss markers of disease progression, reporting on the antigenicity of *Leishmania* antigens and the role of the eukaryotic initiation factors F2, F2B, LieIF2, and LieIF2B. These proteins, to which specific antibodies were detected in the serum of patients with VL, and in dogs with canine leishmaniasis, induce a humoral response in a murine model, along with the production of IL-10. IL-10 favors the progression of the disease and therefore could act as an indicator of the same.

Piel et al. propose experimentally infecting mice with cosmid-transfected parasites as a means of searching for new genetic markers. This might allow the identification of genetic loci associated, for example, with resistance to medications, or that might act as new treatment targets. Any factors thus identified, however, would have to be validated in specific field studies.

## Biomarkers of cure

Some of the articles included in this Research Topic focus on the identification of new biomarkers associated with the response to treatment, and that provide confirmation of cure. A biomarker that indicates a cure to have been achieved could be used to reduce treatment times and prevent relapses, help adjust doses, and be of use in research into new treatments or combinations of current medications (Alves et al., 2018). Marlais et al. report results obtained in a clinical trial involving patients with VL in which they measured *Leishmania*-specific IgG1 in the serum before and after treatment. Using the VL Sero K-SeT rapid diagnostic test and ELISA, they show that high post-treatment concentrations of these antibodies are associated with relapse, while low (or no) concentrations are associated with cure. They also report the above rapid diagnostic test may be of help in the diagnosis of PKDL.

In the search for biomarkers that indicate the cure of CL, Montoya et al. report the use of a hamster model to analyse skin lesions for the production of the growth factors EGF, TGFbeta1, PDGF, and FGF. They indicate an increase in TGFbeta1 to be associated with active disease, while high EGF levels are associated with the cure of the lesion. They conclude that the EGF/TFGbta1 ratio might provide an excellent biomarker of the establishment of an infection or an adequate response to treatment. Kip et al. investigate the role of neopterin, a marker of macrophage activation, and its association with the response to treatment for VL. These authors examine the plasma neopterin concentration before and after treatment and discuss its potential for identifying patients at risk of suffering an early relapse.

Botana et al. compare serological, parasitological and cellular response biomarkers in patients with different forms of leishmaniasis caused by *L. infantum*. Their work, which was performed with patients with active disease plus others who had been cured, shows significant differences in the results of pre- and post-treatment parasitological tests (they became negative), and in cellular immunity tests (they became positive). However, no changes to the outcome of serological tests were seen. These authors conclude peripheral blood mononuclear cell (PBMC) proliferation following stimulation with *Leishmania* antigens, and the secretion of IFN-gamma, to be good markers of cure of VL. However, for CL, MCL, and localized leishmanial lymphadenopathy (LLL), these same tests detected no difference between the active and cured phases.

## Biomarkers of Asymptomatic Infection

Many of the articles in this Research Topic insist on the importance of identifying biomarkers of asymptomatic infection. This is necessary if we are to know the true prevalence of *Leishmania* infection in any determined area, and for designing strategies to control the disease (Alvar et al., submitted). The search for such biomarkers is limited, however, by the deficient definition of an asymptomatic patient as “someone in an endemic area who has an immune response (antibody- or cell-based) against *Leishmania* but who remains healthy.” This partly explains why, to date, there is no reference method for detecting asymptomatic infection.

This collection of articles contains two original research papers that focus on asymptomatic infection. Best et al. report that in asymptomatic persons who had traveled to areas where American tegumentary leishmaniasis (ATL) is endemic, the expression of IFN-gamma following the stimulation of their PBMC with *Leishmania* antigens is directly related to the length of time spent in the area. This can provide information on how long the asymptomatic condition can last.

In very different work, Coutinho-Abreu and Valenzuela provide a comparative phylogenetic analysis of the proteins in sand fly saliva, and report differences in the amino acid sequence of those of New World and Old World flies, and indeed proteins unique to them, that might serve as biomarkers of infection by a determined species.

## Biomarkers for Vaccine Assessment

The search for biomarkers that correlate with the degree of protection achieved are vital in the development of *Leishmania* vaccines (Moreno, 2019). Several contributions to this Research Topic focus on the immune response to the parasite, and on parasite antigens that might be candidates for use in vaccine production. Egui et al. examine the functional and phenotypic profiles of *Leishmania*-specific CD4+ and CD8+ cells from patients cured of CL caused by *L. panamensis*, as well as those of healthy, asymptomatic patients, and report that protection against the disease is associated with an increased cytotoxic T cell response—something that could be very useful when monitoring patients. Boussoffara et al. come to a similar conclusion in their article on CL caused by *L. major*, and report that high levels of granzyme B are associated with better protection against the development of CL. It could therefore be a useful biomarker for assessing the effectiveness of vaccines. As mentioned by Ontoria et al., combining transcriptomic, proteomic, and metabolomic analyses may be of great interest when studying the factors involved in the cellular response, and for identifying biomarkers of infection. The review by Tavares-Veras et al. goes deeper into this question and discusses large scale studies that have identified and assessed biomarkers in infected macrophages. The parasite alters the protein profile of these cells, rendering them suitable for proteomic studies aimed at identifying new molecular biomarkers that might reveal the destiny of the host cell and pathogen (Jean Beltran et al., 2017).

## Biomarkers of Coinfection With HIV and *Leishmania*

Since many of the biomarkers identified are related to the host immune response, others need to be sought for use in situations in which no immune response occurs. Immunodepression increases the risk of developing leishmaniasis, alters the clinical spectrum of the disease, and increases the risk of therapeutic failure and relapse (van Griensven et al., 2014). Biomarkers for monitoring patients coinfected with *Leishmania*, and HIV, and who suffer from some other form of immunodepression, are therefore needed (Akuffo et al., 2018). The present collection contains two articles on the search for biomarkers for the former. In their work, aimed at identifying early markers of susceptibility or resistance to VL in patients with HIV, Adriansen et al. examine the serum levels of macrophage activators (sCD40L and neopterin) in such patients, and confirm that they are reduced in those with active VL, and increased in those with asymptomatic *Leishmania* infection. These markers could be useful when trying to predict the progress of disease in such patients. In contrast, van Griesvan et al. focus on the identification of biomarkers of therapeutic failure for VL and disease relapse in coinfected persons. This research paper shows that coinfected patients with high levels of *Leishmania* antigens in their urine at the moment of diagnosis of VL are at greater risk of therapeutic failure. In addition, those with high levels at the end of treatment are more likely suffer a relapse within 12 months. These results highlight the importance of antigenuria in monitoring the response to treatment and the risk of relapse in immunodepressed patients.

## Conclusions

The present collection of articles underscores the main problems faced in identifying biomarkers of leishmaniasis, and show that much work is needed to validate those already found. It is important that the knowledge we have be used in innovative ways resulting in novel clinical applications and rapid, sensitive and simple diagnostic tests.

## Author Contributions

EC and JM have participated equally in the writing of this editorial.

### Conflict of Interest

The authors declare that the research was conducted in the absence of any commercial or financial relationships that could be construed as a potential conflict of interest.
